# Workload Model Based Dynamic Adaptation of Social Internet of Vehicles

**DOI:** 10.3390/s150923262

**Published:** 2015-09-15

**Authors:** Kazi Masudul Alam, Mukesh Saini, Abdulmotaleb El Saddik

**Affiliations:** 1 Multimedia Computing Research Laboratory, University of Ottawa, Ottawa, ON K1N 6N5, Canada; E-Mail: elsaddik@uottawa.ca; 2 Division of Engineering, New York University in Abu Dhabi, United Arab Emirates; E-Mail: mks4@nyu.edu

**Keywords:** sensing-as-a-service, vehicular sensors, cyber-physical system, Social Internet of Things, Social Internet of Vehicles, vehicular ad-hoc networks, analytical modeling

## Abstract

Social Internet of Things (SIoT) has gained much interest among different research groups in recent times. As a key member of a smart city, the vehicular domain of SIoT (SIoV) is also undergoing steep development. In the SIoV, vehicles work as sensor-hub to capture surrounding information using the in-vehicle and Smartphone sensors and later publish them for the consumers. A cloud centric cyber-physical system better describes the SIoV model where physical sensing-actuation process affects the cloud based service sharing or computation in a feedback loop or *vice versa*. The cyber based social relationship abstraction enables distributed, easily navigable and scalable peer-to-peer communication among the SIoV subsystems. These cyber-physical interactions involve a huge amount of data and it is difficult to form a real instance of the system to test the feasibility of SIoV applications. In this paper, we propose an analytical model to measure the workloads of various subsystems involved in the SIoV process. We present the basic model which is further extended to incorporate complex scenarios. We provide extensive simulation results for different parameter settings of the SIoV system. The findings of the analyses are further used to design example adaptation strategies for the SIoV subsystems which would foster deployment of intelligent transport systems.

## 1. Introduction

Sensing-as-a-service (SenAS) [1] is a new model to describe the Ubiquitous Computing (Ubicomp) or the Internet of Things (IoT) where four conceptual layers are involved from the data provider to the consumption process. In this model, *Sensors* are deployed to collect data about the environment and the sensor owners have the right to publish the sensor services. Here, *Sensor Publishers* (SP) play the broker role such as Internet Service Providers (ISP), where the sensors and their related services are published and any service requests are forwarded through them. *Extended Service Providers* (ESP) offer the intelligent value added services that analyse the consumer requests and search for the sensors and related services that would fit the payment model or the expected quality. *Consumers* are in the final layer of SenAS which ranges from individuals, private or public organizations, to the governments.

The evolution of *Ubicomp* has seen the wave of research trends moving from Wireless Sensor Networks (WSN) to the Internet of Things [2] which eventually created a new paradigm of thoughts Social Internet of Things (SIoT) [3,4]. SIoT views *Ubicomp* as all the *Things* are somehow physically or logically connected and offering their services through the social network of things which is analogous to the social network of humans. The social network of things simplifies the discovery of relevant services and makes *things* easily navigable such as the human social network but with reduced number of physical probes. Though there are some differences in the opinion about the adequate architecture of SIoT (some prefer to use the existing human social network platforms for their availability whereas others see a dedicated social network of *things* is more viable to distinguish their services), there is no doubt about its importance in describing the *Ubicomp*. Again, the integration of domain specific *things* social networks and human social networks is a viable option to bring them in the same platform.

In the age of Smart Cities, vehicles are one of the key elements of the *Ubicomp*. There has been growing research interests for building advanced communication infrastructures for vehicles (e.g., VANETs [5,6,7,8,9]), which is recently trending to the Internet of Vehicles (IoV) [10,11,12]. The authors present a warning generation system in [13] that uses emergency phone towers incorporated with RFID readers or vehicle based smartphones to detect vehicles and collect safety information in a control room for traffic congestion reporting. In [14], the authors describe a three-tier emergency message dissemination system that uses a user-friendly Android app to send roadside events to a central server. The server can send nearby ambulance to the emergency location and also control the traffic lights if required. Tornell *et al.* [15] present a smartphone based easily adaptable Android app that uses eMDR protocol and is interfaced with a navigation system to access road maps, location and route information for emergency message dissemination to selected authorities such as ambulance or police-cars. Balen *et al.* envisioned a VANET simulator [16] where smartphone based VANET nodes will be connected to a central server and peer-to-peer communication will be simulated there. The physical communication from the smartphone to the server will be through 3G/LTE cellular network. A self-organized VANET model named VAiPho was proposed in [17] that does not require physical RSU and all the communications are managed through the smartphones on the vehicles using WiFi-Direct, Bluetooth, and 3G/4G technologies. A popular community-based traffic and navigation mobile app, where drivers share real-time traffic and road info is Waze (https://www.waze.com/).

In our earlier investigations ([18,19]), we have described the vehicular domain of SIoT (*i.e.*, Social Internet of Vehicles (SIoV)) from the theme of machine-to-machine (M2M [20]) social networks where vehicles are the key social elements. As the smart city vision is growing with incredible speed, so the number of vehicles involved in the city life. It has rightfully been predicted that the vehicles along with their sensors will generate enormous amount of interesting data for many stakeholders, which aligns properly with the characteristics of big data: volume, variety and velocity [21].

All the data generated in the vehicles can be aggregated using the standard VANETs techniques. In the research of VANETs, there are three types of data dissemination models: push, pull and hybrid. In the push model, data is broadcasted to a group of vehicles without any specific request. It is generally used for transport safety type applications. The pull model is used for delay tolerant applications (e.g., parking, traffic, gas price or grocery status) where data is only shared in response to a request. A hybrid model combines both of these models according to resource availability and road layout situations [22]. In the SIoV, various services are offered by the physical components either through *ad-hoc* communication or through the 3G/LTE type communication from the virtual or cyber entities. Data dissemination in the SIoV can follow any of the VANETs models.

The new paradigm of SIoT is receiving increased interest from the research community as it perfectly coincides with the social networks, IoT, service oriented architecture and the cloud computing. In the SIoT, the services of the things are navigated using the social network of things or humans but it requires domain specific knowledge, e.g., vehicle specific design decisions are required to build SIoV. In this paper, we study the effect of data workloads on individual system components that are involved in a SIoV and how traffic parameters affect them. The models developed in the study are important to dynamically manage the workloads over cyber-physical subsystems so that the SIoV can perform accordingly at different scenarios. SIoV offers a new approach to tackle the intelligent transport system (ITS) related issues when number of vehicles are increasing dramatically and most of the technologies are embracing more of cloud computing and service oriented architecture.

The contributions of this paper are the analytical models to estimate the workloads of different subsystems of the SIoV. We validate the proposed workload models using extensive simulations and measure the effects on the workloads at different system settings. This allows us to find the relationships among the system parameters, which are used to design example adaptive algorithms for the subsystems to adjust the overall system goal. The proposed model of SIoV adheres to the demonstrated knowledge of VANETs which allows easier adaptation to the established standards. The rest of the paper is organized as follows. Section 2 discusses related works, followed by an introduction to the SIoV in Section 3. Later, Section 4 elaborates the basic scenario modeling which is further extended to adopt other complex scenarios in Section 5. Simulation results and related analyses are presented in Section 6. Example adaptive algorithms for the subsystems are discussed in Section 7 followed by conclusion and future works in Section 8.

## 2. Related Works

Vehicular communication technology is growing rapidly. A large body of researchers are working on the design and development of the communication standards and related routing protocols. As the communication technology is maturing, we witness research works on information dissemination over the vehicular networks. Consequently, SIoV is emerging as a promising application of big data from the IoT philosophy where vehicular sensor data will be consumed by different types of clients through the social network of services. There are few works related to VANETs traffic or safety message dissemination modeling from communication and routing perspective. In this paper, we propose application centric workload models of SIoV subsystems which will be helpful to build SOA, cloud computing or social network based IoV applications.

In [23], the authors develop a finite queue model to analyze queue occupancy distribution at junctions and traffic signals. They measure waiting time distribution and traffic congestions to find shortest path and receive early jam alert. In [24], the authors propose an approach to avoid broadcasting storm in VANETs by prioritizing a subset of locally generated messages over distant messages for the safety applications. In their model, they attempt to minimize the critical distance between the event location and the vehicle that would receive and react upon the event information.

In [25], the authors modeled connectivity in the vehicular *ad-hoc* network in highway scenario. They modeled traffic states in terms of vehicle speed, traffic density and traffic flow. The authors first modeled the connectivity using platoon size and connectivity distance metrics and later extended the basic model for complex scenarios such as two direction traffic, multilane highways, and heterogeneous vehicle networks. Their analysis shows that the increase of traffic flow and transmission range is good for connectivity whereas higher speed reduces connectivity. Since IoT is increasingly becoming a Sensing-as-a-Service model, we study the mathematical model of [26] that measures the performance of web service composition. Their proposed model can predict web service utilization changes, the duration to complete new process calculations, as well as optimize the *Service Level Agreements* of the service providers and the service integrators.

Again, from a data perspective, safety messages dissemination about a hazardous condition was studied in [27], where the authors measure the performance in terms of average delay to propagate the message, the number of nodes receiving the relay information and the number of duplicate messages received by each vehicle. Data dissemination algorithms with time-probabilistic characteristics are described in [28], where the authors provide a simplified model of the dissemination delay. In [29], the authors provide analytical study of the performance of data dissemination in VANETs. They divide the traffic in two priority classes, higher priority and lower priority. The performance is measured in terms of average message forwarding distance. Their analysis proves that the increase of transmission range improves message forwarding but the improvement is limited due to internal and external interferences at the receiver node.

In our previous works, we first [18] introduced and later [30] detailed the architecture and interactions of machine-to-machine social networks for the IoV (*i.e.*, SIoV) which aligns with the research challenges envisioned in [31] for ITS. In this paper, we present the workload analysis of various subsystems involved in the SIoV process to identify the contextual relationships so that the system can dynamically adapt and reconfigure its settings according to the expected goal. This is an important property for the future ITS applications. The model would help to measure the workload of cloud computing, service oriented architecture and social network based IoV applications that would foster real life deployments.

## 3. Social Internet of Vehicles

**Figure 1:**
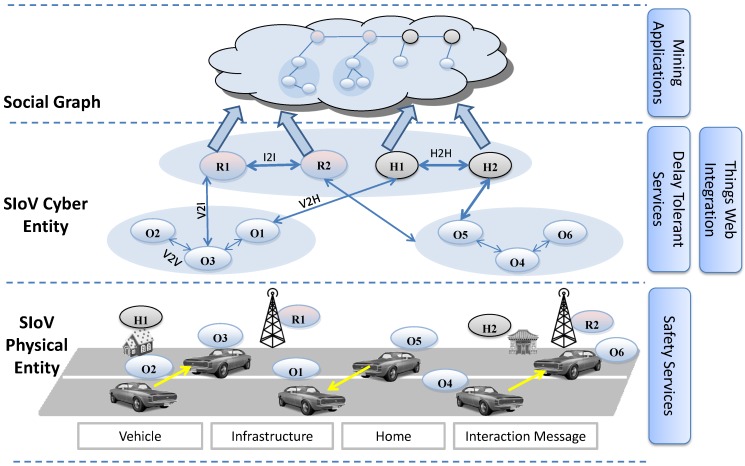
Architecture of Social Internet of Vehicles.

The Social Internet of Vehicles (SIoV) (Figure 1) is a type of cloud based cyber-physical system [32,33] where physical systems involved in VANETs have virtual entities, similar to the virtual sensors of global sensor network [34]. This middleware based cyber or virtual computing maintains one-to-one feedback loop with the physical processes. Service computing or sharing affects physical sensing or actuation and *vice versa*. The cloud based cyber entities enable virtual peer-to-peer networking which fosters heavily decentralized cloud operation (e.g., fog computing [35]). SIoV uses cyber-physical abstraction to form machine-to-machine (M2M) social network in the IoV domain to offer services related to safety, efficiency, comfort and entertainment applications. The main physical components (*i.e.*, SIoV subsystems) involved are vehicles (V), roadside infrastructures (I), houses (H) and the cloud (C). These physical components also have their representative virtual entities. SIoV follows SenAS model where components such as *V/I/H* offers services related to real time or delay tolerant applications and *C* offers knowledge driven data mining applications. Every physical or virtual component has its unique ID and works as a hub for the sensors and services. The presence of cyber or physical entity in communication is ubiquitous to the consumers. The system takes smart decisions between physical (*i.e.*, *ad-hoc*) or cyber (*i.e.*, 3G/LTE) communication based on the current scenario or demand.

SIoV assumes that a physical vehicle is capable of establishing WiFi *ad-hoc* (e.g., VANETs) connections with other neighbour vehicles while being connected to the internet using 3G/LTE type communication technology [10]. Based on the SenAS model, the sensory data or the services are first provided by the ad-hoc wireless channel or, if not possible, from the virtual entity through the cloud. As a result, the services are omnipresent and network communication is ubiquitous. The types of interactions where data is generated or aggregated and the service requests are initiated are vehicle-to-vehicle (V2V), V2I, I2I, I2C, V2H, H2H, and H2C. Possible service responses are V2V, I2V, I2I, and H2V. We consider virtual entities synonymous to physical *things* as the decision of getting services from either is invisible to the sensors or services consumer.

The SIoV has two types of data sharing scenarios [18,30], (1) *Static*: a vehicle is contacting with its home or with its physical friends to share data using V2H or H2H, (2) *Dynamic*: a vehicle is moving on the roadway and sharing information with other moving vehicles and the road side infrastructures using V2V or V2I. Dynamic relationships are temporary and tracked using time stamp, whereas static relationships are more lasting. Static friends are those which maintain lasting relationships with the owner of the vehicle. For example, the neighbour houses where the vehicle owner lives or the mechanic shop where he takes his vehicle for servicing or the manufacturer of the vehicle are static friends. Data shared among the static friends are more private than the dynamic data exchanges that occur in the public infrastructure. Cyber SIoV is an abstraction of the physical systems where all the existing or current SIoV relationships are managed.

It has been recommended in VANETs literature for the vehicles to emit “*i am here*” type messages 10 times/s [36] for safety applications. Other types of interaction messages are infrequent and they only occur based on specific requests from other vehicles. Every physical vehicle along with its current sensory information are also accessible through the cyber layer of relationships. Cyber entities maintain their relationships using peer-to-peer connections. The services offered by every vehicle can be consumed either from the physical vehicle or through corresponding cyber entity. Services can be free of cost or can have a price tag agreed among the consumers and the providers. The interactions among the SIoV subsystems are managed as a social graph. In the social graph, nodes are the participating physical subsystems (*i.e.*, V/I/H) and the edges are the relationships and interactions. Important interaction information are stored in the edges with their respective time stamp. Details about social graph data structure can be found in [30].

In this paper, we assume that OBU, RSU and HBU are the corresponding devices that store the social graph data of the vehicle (V), infrastructure (I), and the house (H) respectively. While a vehicle is travelling from one roadside infrastructure to the other, it generates sensory messages to be shared with neighbouring vehicles (*i.e.*, platoon members). Important V2V sensory messages are stored in the receiving vehicles in a graph like data structure that is called OBU-OBU social graph. Over time, one vehicle passes through many different platoons and observes many V2V interactions. As a result, the OBU-OBU social graph of a vehicle grows and becomes bulky. When a vehicle that is storing the OBU-OBU social graph gets into the communication range of the infrastructure, it tries to hand over the social graph to the infrastructure using V2I interaction. If this operation is successful then the transferred OBU-OBU social graph is appended with OBU-RSU social graph of the infrastructure. An infrastructure can receive many OBU-OBU social graph over time, which are added as subgraph to the OBU-RSU social graph. In case of an incomplete V2I handover, the transaction is completed in the following infrastructure which is later synchronized to the originating infrastructure using I2I interaction. If no infrastructure is found then the transaction is completed in a house (e.g., own or friend) using V2H interaction which leads to OBU-HBU social graph.

All the momentary OBU-HBU or OBU-RSU social graph data available in a house or in an infrastructure of a particular region are transferred to a representative cloud, which leads to a cloud based OBU-RSU-HBU social graph. As the social graph is both time and location stamped hence the cloud can reorganize the fragmented subgraphs to their appropriate positions over time. Location and time are important attributes in the social graph. Since wireless communication is omnipresent and data can be duplicated, hence every physical component (*i.e.*, SIoV subsystem) puts effort to clean the respective redundant subgraph. The layered social graph of vehicle, infrastructure, house and the cloud enables with different sets of application. Here OBU-OBU social graph contains information for real time safety applications, whereas OBU-RSU social graph provides delay tolerant services and cloud graph can be used for data mining. Virtual social networks of the physical *things* represent the relationships of the *things* at any time and the social graph represents their degree of interactions. The former is a overlay peer-to-peer network on the physical *things* and the later is somewhat persistent interaction data.

**Table 1:** List of symbols.

Symbol	Description
*v*	Average speed of the vehicle
*L*	Distance between two Infrastructures
*T*	Time required for a vehicle to reach next *I* with *v* speed
*s*	Sensor sampling rate
λ	Data rate of information type
$r_{v},r_{i}$	Communication range of vehicle and infrastructure
$n_{h},n_{a}$	Number of home friends and number of homes
*d*	Sample size
*D*	Data workload
*f*	Data fraction function
i,j,k	Numbering parameters
p(x)	probability of a successful event *x*
*e*	Occurrence of an event
ψ	Storage requirements of a subsystem

## 4. The Basic Model

**Figure 2:**
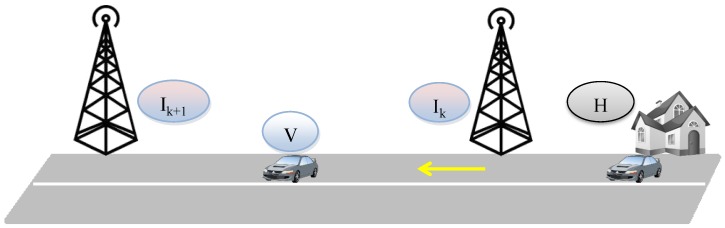
Scenario of the basic Social Internet of Vehicles (SIoV) model.

We take an incremental approach to model SIoV; first we model the basic scenario and then we gradually extend the basic model to incorporate more complex scenarios. The basic scenario (Figure 2) consists of a vehicle that belongs to a household and travels from the infrastructures $I_{k-1}$ to $I_{k}$ on a one way road. The vehicle offers services related to safety, efficiency, comfort, and infotainment. As there are no other vehicles offering their services, no sharing occurs; the sensory information generated by one vehicle is stored in its OBU-OBU social graph. When this vehicle reaches the infrastructure $I_{k}$, it hands over the data to it and $I_{k}$ builds OBU-RSU social graph. After a predefined time, the infrastructure transfers the OBU-RSU social graph to the cloud. Following sections describe the workload models for different SIoV system components in this setup. In the proposed system model, workload is measured in terms of data storage requirements for the SIoV subsystems in a time period *T*:

- Vehicle data storage ($\psi_{v}$) is used to store OBU-OBU social graph that is composed of sensory information and V2V interactions.
- Infrastructure data storage ($\psi_{I}$) is used to store OBU-RSU social graph that is generated in V2I or I2I interactions.
- Home data storage ($\psi_{h}$) is used to store incomplete V2I and H2H interaction data.
- Cloud data storage ($\psi_{c}$) is required to aggregate regional or residential infrastructure and home data.

### 4.1. Assumptions

**Figure 3:**
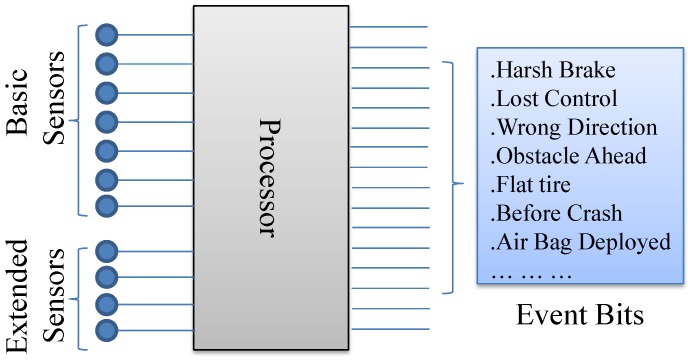
Sensory data to events identification.

We assume that the vehicle is travelling at a constant speed (*v*) in between the infrastructures that are *L* distance apart, there is no traffic jam, the traffic is one way and each vehicle has $r_{v}$ communication range. Every vehicle is equipped with basic and extended sensors. Basic sensors are used to generate public information for safe manoeuvring (e.g., VANET “i am here” message). On the other hand, extended sensors generate intermittent messages that describe events occurring in and around the vehicle (e.g., sharp brake, lost control, obstacles ahead, wrong direction, *etc*.) (Figure 3). In addition, messages also contain the interaction information (e.g., efficiency, comfort, entertainment requests) which is different from the requested content.

### 4.2. Event Definition

Every type of service requests are considered as events. Events can also be detected by processing the sensory data. All the event occurrences are represented using a fixed bit and (on/off) value (Figure 3).

In case of regular “*i am here*” type message, only the basic sensory data are shared with the surrounding vehicles. When extended sensors detect an event, all the sensory data (*i.e.*, basic + extended) including representative event bits are also shared with the neighbouring vehicles either through physical connection or through virtual connection. In an event byte, a checked bit represents an event occurrence. Multiple checked bits in a byte represent multiple event detections. For example, 16 and 32 event bytes can represent individually 16 × 8 (bits) =128 and 32 × 8 (bits) = 256 events respectively.

### 4.3. Interaction Data

In the SIoV, the messages generated by vehicles consist of two parts, dynamic and static. Static part is of fixed size and includes vehicle or user related administrative information such as vehicle number or user details. Dynamic part consists of indoor and outdoor sensory data, and other social service requests related to comfort, efficiency, and entertainment. The service requests can use physical ad-hoc communication range, or use virtual communications if out of physical range through 3G type connections.

If $n_{b}$ and $n_{x}$ are the number of basic and extended data sensors respectively deployed in a vehicle, *s* is the sampling rate of a sensor, and *d* is the sample size, then the vehicular sensory data generation rate can be

(1) $$\text{Basic data rate},\lambda_{b}=\sum_{i=1}^{n_{b}}\left(s_{i}*d_{i}\right)$$

(2) $$\text{Extended data rate},\lambda_{x}=\sum_{j=1}^{n_{x}}\left(s_{j}*d_{j}\right)$$

The static information about a vehicle rarely changes over the time such as vehicle identification (e.g., serial number, manufacturer, model), physical attributes (e.g., length, height, weight), vehicle exterior (body, axles, frame), *etc*. This information is synchronized to the cloud through home (H), when a new vehicle joins to the SIoV. This information represents both the physical and the virtual vehicle. If $\lambda_{sv}$, $\lambda_{dv}$ are the data rate of static and dynamic vehicular information respectively then total vehicular data rate $\lambda_{v}=\lambda_{sv}+\lambda_{dv}$ where $\lambda_{dv}=\lambda_{b}+\lambda_{x}$.

Again, user descriptive message has two parts: (1) static information such as name, ID, license number, age, *etc*; (2) dynamic information that changes over time such as physiological parameters and mental state obtained through sensors [30]. A new user (*i.e.*, driver) of SIoV shares his private static information ($\lambda_{su}$, data rate) through the home. Whereas, the public dynamic user information ($\lambda_{du}$, data rate) is shared with other vehicles and infrastructures. Here, $\lambda_{u}=\lambda_{su}+\lambda_{du}$.

If $du_{e}$ is the number of dynamic user status entities, $n_{e}^{u}$ is the number of properties in an entity, $d_{ij}$ is the size of *i*th entity’s *j*th property, and $s_{du}$ is user status sampling rate, then $\lambda_{du}$ can be

(3) $$\lambda_{du}=s_{du}*\sum_{i=1}^{du_{e}}\sum_{j=1}^{n_{e}^{u}}d_{ij}$$

Here, $\lambda_{su}$ and $\lambda_{sv}$ contributes to static type data and $\lambda_{du}$ and $\lambda_{dv}$ produces dynamic data. Also, other dynamic data requests are generated from efficiency, comfort and entertainment type requests whose data rates are $\lambda_{ef}$, $\lambda_{cm}$, and $\lambda_{en}$ respectively.

### 4.4. Performance Measures

We consider two types of interactions in the SIoV: push and pull. In the push type interactions, data is shared without any specific request from others, such as safety information sharing. These interactions are given highest priority and data can travel in any path physical or virtual whichever way is the most efficient. On the other hand, pull type interactions take place in response to the requests from others, such as request for traffic status, parking information or entertainment media sharing, *etc*. The type of the interactions are used to describe the actions of the system, they do not have effect on the model design. Following sections describe the performance measures of the basic scenario case.

#### 4.4.1. Vehicle Data Storage ($\psi_{v}$)

**Figure 4:**
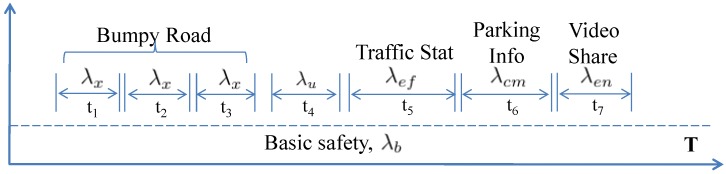
An example timeline of vehicle storage, $\psi_{v}$ in T time.

We consider the time a vehicle takes to travel from infrastructure $I_{k}$ to $I_{k+1}$ as *T* and all the successful interactions (e.g., V2H, V2V, V2I) as vehicle related events. Figure 4 shows how different types of interactions take place over *T* time. Basic safety information is continuously shared with a data rate of $\lambda_{b}$. Extended safety information (e.g., Bumpy road) is only shared during few time intervals with an additional data rate of $\lambda_{x}$. Also, the dynamic user information can be shared intermittently at $\lambda_{du}$ rate. Similarly, efficiency, comfort and entertainment message requests incur $\lambda_{ef}$, $\lambda_{cm}$ and $\lambda_{en}$ data rates respectively during different time intervals. A vehicle stores all the data generated by itself (push interactions) and data received during social (pull) interactions. If $D_{g}$ indicates the data generated and $D_{r}$ represents the data received, then the total data of any subsystem is $D=D_{g}+D_{r}$. The data storage required ($\psi^{v}$) by a vehicle while travelling between two infrastructures and only generating sensory observations (not receiving) can be represented as,

(4) $$\psi_{v}=\lambda_{b}*T+\sum_{i=1}^{n_{e}}\left(\lambda_{i}*t_{i}\right),\text{where},\lambda_{i}\in \left\{\lambda_{x},\lambda_{du},\lambda_{ef},\lambda_{cm},\lambda_{en}\right\}$$

Here $n_{e}$ is the total number of interactions, $\lambda_{i}$ is the data rate corresponding to *i*th interaction type; and $t_{i}$ is total duration of *i*th type of interaction. The time required for each type of event can be different based on the communication range and vehicle speed. Safety like push based data generation is considered mandatory and other pull based interactions are optional for the provider vehicle. Note that in the basic scenario, where there is only one vehicle, there are no pull type interactions (*i.e.*, $\lambda_{ef}=\lambda_{cm}=\lambda_{en}=0$). Also, in this model we are only considering dynamic information. Because, the overhead of storing static information ($\lambda_{sv}$ and $\lambda_{su}$) is negligible in comparison to the dynamic data.

#### 4.4.2. Infrastructure Data Storage ($\psi_{I}$)

**Figure 5:**
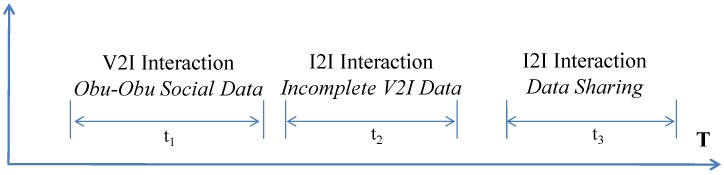
An example timeline of infrastructure storage, $\psi_{I}$ in T time.

In the basic scenario, we have only one vehicle passing through the infrastructures. Hence, it is part of a platoon of one member and all the data generated while travelling will be transferred to the infrastructure ($I_{k}$). As mentioned earlier, if the vehicle is unable to transfer all the data, then the remaining data is transferred to the next infrastructure in line (e.g., $I_{k+1}$). The next infrastructure sends the data back to the originating infrastructure through I2I interaction. In addition, the infrastructure may also receive other types of I2I interaction requests (Figure 5) from the neighbour infrastructure. If we consider the interactions in *T* time then $\psi_{I}^{k}$ can be represented as,

(5) $$\psi_{I}^{k}=\psi_{v}+f_{I}\left(\psi_{I}^{k-1},\psi_{I}^{k+1}\right)$$

where $\psi_{v}$ is the data contributed by a vehicle and $f_{I}$ returns the shareable data of the right (k+1) and left (k−1) neighbours of any infrastructure. The number of neighbours can be more than two based on the shape of the road layout and how many different traffic directions approaching the infrastructure.

#### 4.4.3. Home Data Storage ($\psi_{h}$)

Every vehicle belongs to a household where it shares its private information or uncommitted V2I or V2V data using V2H interactions. Static user data ($\lambda_{su}*t_{2}$) and static vehicular data ($\lambda_{sv}*t_{1}$) are also shared through the V2H interactions where $t_{1}$, $t_{2}$ are the time to complete respective transactions. Moreover, services can be shared with the local or remote friends using H2H interactions. But in basic model we consider only one house, hence the data storage requirements for the home is

(6) $$\psi_{h}=f_{h}\left(\psi_{v}\right)$$

Here $f_{h}$ is the function that provides the uncommitted vehicular data fragment of the residence vehicle.

#### 4.4.4. Cloud Data Storage ($\psi_{c}$)

All the data collected in the infrastructure and the home are committed to the cloud. Hence, for the basic model of SIoV, cloud storage requirements can be depicted as

(7) $$\psi_{c}=\psi_{I}+\psi_{h}$$

## 5. Extension of the Basic Model

**Figure 6:**
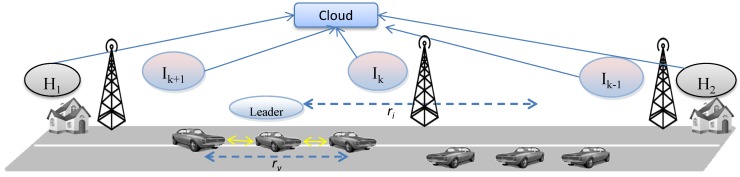
A scenario of the extended model.

The basic model describes the SIoV scenario where one vehicle belongs to a household and travels from one infrastructure to the next infrastructure. We further extend the basic model to, (1) incorporate more vehicles to the dynamic scenario so that the vehicles can form platoons of multiple members; (2) introduce a region with multiple infrastructures so that inter-infrastructure communication can be logged; (3) add more houses to the residential areas to extend the number of local and remote friends (Figure 6). Following sections describe the models in detail.

### 5.1. Multiple Vehicles Scenario

**Figure 7:**
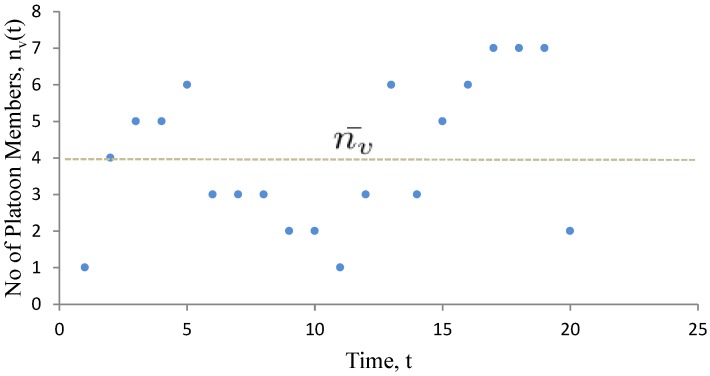
In the extended model, a vehicle becomes member of multi-member platoons over time and will receive sensor data from many neighbour vehicles.

In the multiple vehicles scenario, while moving between the infrastructures, a vehicle becomes part of multi-member platoons (Figure 7) over time based on its communication range. In a multi-member platoon, all the vehicles generate ($D_{g}$) their sensory observations as well as share ($D_{r}$, V2V interaction) them with the other members. So, the number of vehicles interacting at any time can be represented as $n_{v}\left(t\right)$. In the practical scenario, $n_{v}\left(t\right)$ depends on the traffic arrival rate, vehicle speed, traffic density and the communication range. The data generated and received by any vehicle in a multi-vehicle scenario can be represented as,

(8) $$\psi_{v}^{\prime }=D_{g}+D_{r}\to \psi_{v}+\frac{\psi_{v}}{T}*\int_{0}^{T}\left(n_{v}\left(t\right)-1\right)\;dt\to \psi_{v}*\bar{n_{v}},\text{where},\bar{n_{v}}=\frac{1}{T}*\int_{0}^{T}n_{v}\left(t\right)\;dt$$

Here, $\bar{n_{v}}$ is the average number of vehicles that are involved in message sharing at any time. Equation (8) shows that the number of vehicles in a platoon can change at every time step. Here, the connection life of any platoon depends on the communication range and the speed of the vehicles. If $t_{p}$ is the average time for which each platoon connectivity lasts then $n_{p}=\frac{T}{t_{p}}$ is the number of platoons available in between two infrastructures.

Since the interaction can go through the physical layer or the cyber layer and wireless communication is omnidirectional, so we can assume a vehicle will receive most of the messages delivered in the platoon. If one vehicle is selected as a platoon leader at every time, then the platoon leader can only share the data with the infrastructure.

### 5.2. Multiple Infrastructures Scenario

**Figure 8:**
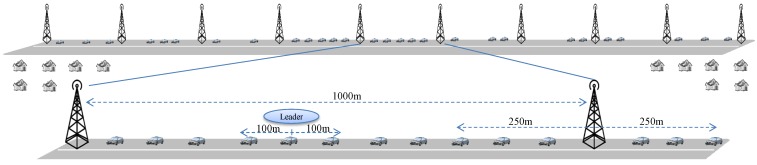
A detailed scenario setup of the extended model.

After introducing more infrastructures to the last scenario, the existing model gets extended to a region of infrastructures (Figure 8). In this case, we have more V2I and I2I interactions in addition to more V2V interactions. In case of V2I interactions, once a vehicle reaches the range of an infrastructure, it uses *push* method to share the OBU-OBU social graph. We assume the number of vehicles approaching an infrastructure in the one-way scenario per unit of time (e.g., a second) is a Poisson process with mean $\frac{1}{\rho }$ [25]; each infrastructure has a communication range of radius $r_{i}$; and it is operating for *T* time. So the workload of an infrastructure in *T* time can be regarded as

(9) $$\psi_{I}^{{}^{\prime }k}=\rho *T*\left\{\psi_{v}^{\prime }+f_{I}^{\prime }\left(\psi_{I}^{{}^{\prime }\left(k-1\right)},\psi_{I}^{{}^{\prime }\left(k+1\right)}\right)\right\}$$

Here, $I_{k}$ can receive the incomplete V2I exchange data from $I_{k-1}$ and other request can come from both $I_{k-1}$ and $I_{k+1}$ through I2I interactions. $f_{I}^{\prime }$ is a function whose parameters are all the infrastructures that are directly connected to *k*th one and an algorithm decides how much data of each neighbour will be shared. The algorithm will adapt to scenarios with two way traffic as well as different road layouts. In this model, we are considering each infrastructure has two neighbours and they are placed in a string. In a multi-way traffic, the workload can be multiplied by the number of vehicular directions approaching, $\psi_{I}^{{}^{\prime \prime }k}=n_{d}*\psi_{I}{}^{\prime }k$, where $n_{d}$ is the number of directions [25].

With more infrastructures, we get a dynamic geographical region where a string of infrastructures are placed and vehicle platoons move from one block to the next infrastructure block. Lets assume, there are $n_{I}$ number of infrastructures in a region, hence the workload of a particular region in *T* time is

(10) $$\psi_{reg}^{T}=\sum_{k=1}^{n_{I}}\psi_{I}^{{}^{\prime }k}$$

### 5.3. Multiple Homes Scenario

**Figure 9:**
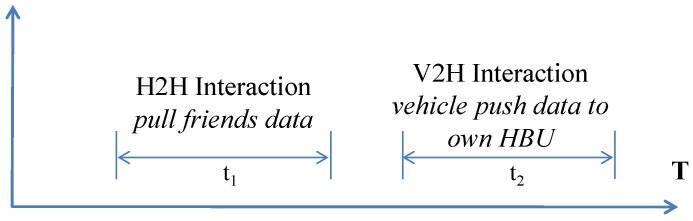
Event timeline of Home Base Unit (HBU) storage, $\psi_{h}$ in T time.

In case of multiple homes scenario, static friends regions grow, which offer greater level of V2H and H2H interactions. In the V2H interaction, one vehicle uses push to send usage data to its corresponding HBU storage ($\psi^{h}$). Whereas in H2H interactions, a home can share data with static friends using pull request (Figure 9). We assume that a home stores a fraction of the data which is collected by a vehicle in *T* time, $p(e_{h})$ is the probability of a successful (*i.e.*, a pull request is answered) H2H interaction and $n_{h}$ is the number of requests a home makes or receives. Then the workload of a house can be represented as

(11) $$\psi_{h}^{\prime }=f_{h}\left(\psi_{v}^{\prime }\right)+p\left(e_{h}\right)*\sum_{k=1}^{n_{h}}f_{h}^{\prime }\left(\psi_{v}^{{}^{\prime }k}\right)\to f_{h}\left(\psi_{v}^{\prime }\right)+p\left(e_{h}\right)*\sum_{k=1}^{n_{h}}f_{h}^{\prime }\left(\psi_{v}^{{}^{\prime }k}\right)$$

Here, $f_{h}$ and $f_{h}^{\prime }$ deliver the data fragments of the vehicle and interaction with static friends respectively. If $n_{a}$ is the total number of homes available in a SIoV instance then the residential workload can be regarded as

(12) $$\psi_{res}^{T}=\sum_{k=1}^{n_{a}}\psi_{h}^{{}^{\prime }k}$$

### 5.4. Social Graph Data Cloud Workload

All the data that are collected in the home or infrastructure are transferred to the social-graph cloud after a certain time. Home operation time is selected by the owner of a vehicle and the infrastructure time is selected by the SIoV provider of a region. For a region of infrastructures and residents, the total workload of the SIoV cloud in T time can be represented as

(13) $$\psi_{c}^{T}=\psi_{reg}^{T}+\psi_{res}^{T}$$

If the time period is $t_{1}$ to $t_{2}$ then the cloud workload can be

(14) $$\psi_{c}^{t_{1},t_{2}}=\frac{\left(t_{2}-t_{1}\right)}{T}*\left(\psi_{reg}^{T}+\psi_{res}^{T}\right)$$

## 6. SIoV Characteristics Analysis

In this section, we study the system workload behaviour using extensive simulation and modeling results for the different parameter settings of the SIoV system. Based on the study, we identify the relationships among the system parameters and the sub-system workloads, which are later used to design adaptation techniques for the SIoV system.

### 6.1. Simulation Setup

**Figure 10:**
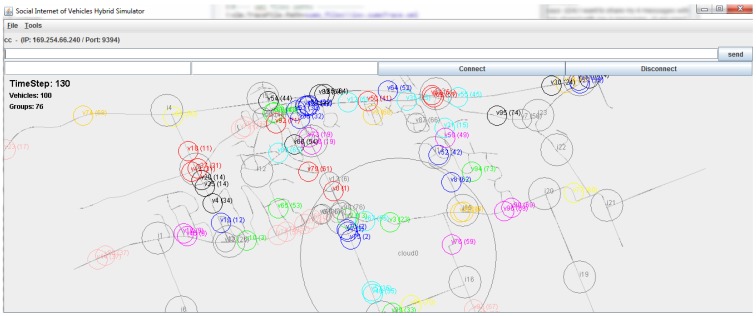
In this simulation window, we present 100 simulated vehicles, 25 infrastructures and 1 social graph cloud on the Cumberland, Ottawa, Canada region. In this picture, i(x) represents infrastructure process, v(x) represents vehicle process, cloud0 represents social cloud process, and same colored v(x) vehicles are connected in a platoon as v(x)(y).

We have designed and developed a simulator (Figure 10) that uses OpenStreetMap (https://www. openstreetmap.org) for map area selection, SUMO [37] to generate mobility traces on the map, JAVA to generate virtual sub-system processes and sockets for peer-to-peer network communication. The map data can further be augmented with sub-system (*i.e.*, infrastructure, home, cloud) details. For the simulation measurement, at first medium scale vehicle mobility traces are generated using SUMO which are further customized by changing the simulator parameters such as vehicle arrival rate (ρ), and vehicle speed (*v*). Later, socket based network communication among the sub-system processes are managed by a central process by using the vehicle communication range ($r_{v}$), inter infrastructure distance (*L*), and the RSU communication range ($r_{i}$) parameters. In our simulation implementation, every physical element (*i.e.*, vehicle, infrastructure) is represented using a virtual process that can engage in peer-to-peer inter-process communication while running in the cloud. The network communication module of the simulator uses the geographical information from the mobility traces and apply communication parameters so that the virtual elements can establish ad-hoc or static network connections and exchange JSON (Javascript Object Notation) data among them. We consider the network connections to be error-free and data generation rate (λ) of 512-to-1024 bytes/s. All the generated and shared data are stored in the respective MySQL database of every virtual element. Once a virtual vehicle reaches the communication range of a virtual infrastructure, it establishes network connection between the virtual processes and later hands over the data to the infrastructure. The implementation details of the simulator architecture is out of scope of this article and will be detailed in a future article. In our simulation setup, arrival rate (ρ) varies from 0.4 to 2.0, vehicle speed varies from 60 Km/h to 140 Km/h, vehicle communication range ($r_{v}$) varies from 50 m to 350 m, infrastructure density varies from 500 m to 5000 m. For each simulation scenario, we collect data for 100 executions and report their average.

### 6.2. Relationship of Subsystem Storage Requirements

**Figure 11:**
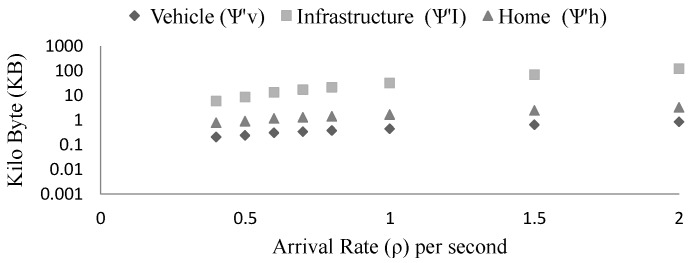
The storage requirements of vehicle, infrastructure and home at various arrival rate (ρ) based on the model. Y-axis is measured in log scale of base 10 (thousands unit).

From the Figure 11 we see the data relationship of different sub-systems. Since vehicle is the only data generator hence the storage requirements of the infrastructure and the home are proportional to the vehicle (*i.e.*, $\psi_{I}\propto \psi_{v}$ and $\psi_{h}\propto \psi_{v}$ ). For this test, we consider v = 16.67 m/s, L = 1000 m, $r_{v}$ = 200 m, $r_{i}$ = 500 m, $n_{h}=20$, and arrival rate ρ is varied. Vehicle arrival rate (ρ) 0.5 denotes that one vehicle is arriving to the scenario in 2 s. With the mentioned setup, the platoon size grows with the increment of arrival rate. For example, at ρ=0.4 platoon size is 5 and at ρ=2 platoon size is 24. Platoon size (P) is the number of vehicles that are connected to exchange information at any time.

**Figure 12:**
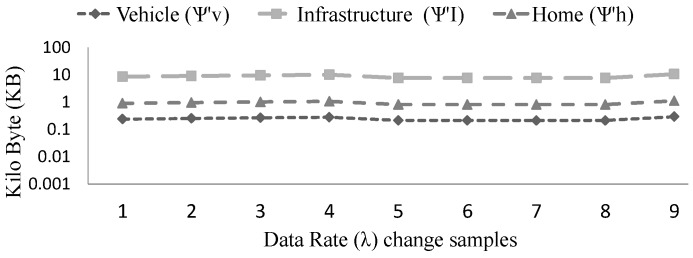
Comparing the storage requirements of vehicle, infrastructure and home on the change of data rate (λ). From sample 5 to 8, safety information is not stored. Y-axis is measured in log scale of base 10 (thousands unit).

Again, from the Figure 12 we see that with the increase of data rate the storage requirements grow and with the decrease of the data rate, storage requirements diminish (ψ∝λ). For example, if we decide to store all the generated safety messages then the storage requirements grow but if we only store the safety messages when there is any important event then the storage requirements decrease. As a result, we can control the workload of the overall system by storing the valuable information only.

### 6.3. Effect of Arrival Rate (ρ)

**Figure 13:**
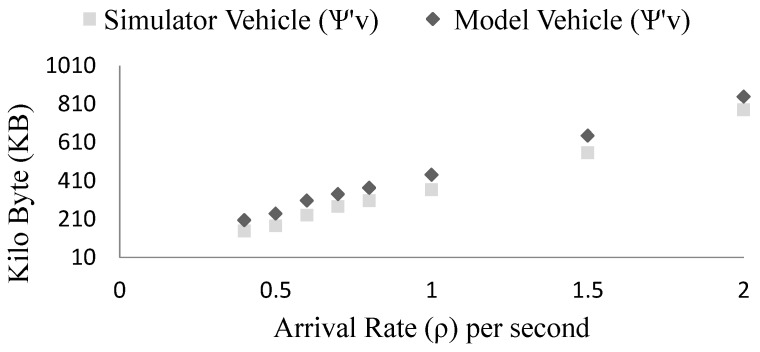
Comparison of the simulation and the model based vehicle storage requirements at different arrival rate (ρ).

In the Figure 13, we compare the storage requirements of vehicles based on simulation results and the modeling results. In this case, we consider v = 16.67 m/s, L = 1000 m, $r_{v}$ = 200 m, $r_{i}$ = 500 m, $n_{a}=20$ but ρ is varied. The simulation results are very close to the modeling results because both the model and simulation considers average number of platoon members. With the increase of arrival rate the storage requirement also grows (ψ∝ρ).

### 6.4. Effect of Vehicle Speed (*v*)

**Figure 14:**
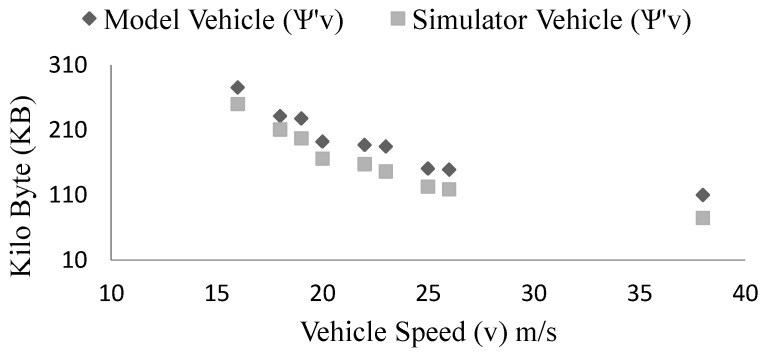
Comparison of the simulation and model measurements of vehicle storage requirements in case of varying vehicle speed (*v*).

In case of vehicle speed effect, we keep arrival rate to ρ=0.5, vary the vehicle speed from 60 km/h = 16.67 m· s${}^{-1}$ to 140 km/h = 38.89 m· s${}^{-1}$ and keep intact the rest of the parameters as described in the earlier section. We observe from the Figure 14 that the storage requirements decrease with the increment of speed ($\psi \propto \frac{1}{v}$). It is to be noted that with higher *v* measurement time *T* reduces. From the figure we see that the model results are very close to the simulation results. With the increment of speed, the connection life of vehicles reduce. As a result, the platoon members interacting at any time falls which reduces the interaction data. In a scenario where vehicles are slowing down due to slow traffic onwards while keeping the same arrival rate and vehicle communication range, the storage requirements surge. When, the speed is almost zero (0.25 m/s) the maximum platoon size grows to around 400.

### 6.5. Effect of Communication Range

**Figure 15:**
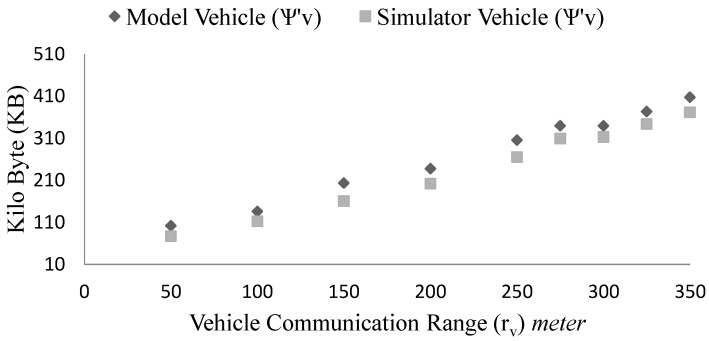
Effect of vehicle communication range ($r_{v}$) on the vehicle storage requirements in case of simulation and the model.

From the Figure 15, we see that with the increase of communication range ($r_{v}$) of the vehicles the storage requirements grow ($\psi \propto r_{v}$). This is because, more vehicles join the platoon and exchange information. As a result, each vehicle listens to more of their neighbours. In the given setup (ρ = 0.5, v = 16.67 m · s${}^{-1}$), where $r_{v}$ is changing from 50 to 350 m, platoon size grows from 2 to 11. If we also vary the infrastructure communication range ($r_{i}$) then for higher communication range, more vehicles will be able to report to the infrastructure at any time. The V2I connection time would be longer in this case. The modeling result is very close to the simulation result as well.

### 6.6. Effect of Infrastructure Density (*L*)

**Figure 16:**
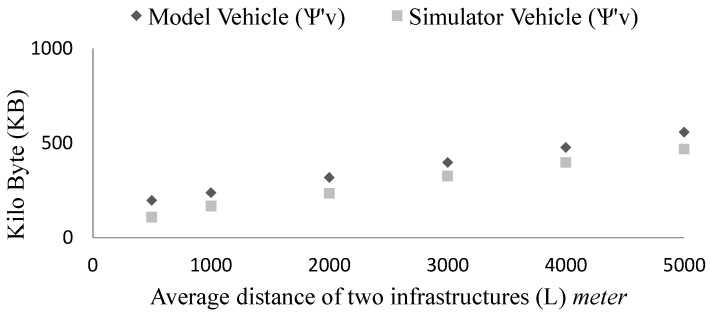
Effect of roadside infrastructure density (*L*) on the storage requirements for the simulation and the model.

When we vary the distance of two consecutive infrastructures, the storage requirements change. From the Figure 16 we see, when the distance grows the storage requirements also grow (ψ∝L). As the arrival rate (ρ=0.5) is unchanged and the vehicle speed is also same v = 16.67 m/s, so each vehicle at a time interacts with 5 other neighbours but they travel for longer time to report to the upcoming infrastructure. As a result, $\psi_{v}$ grows over time.

## 7. Dynamic Adaptation Strategy of the SIoV

Characteristics analysis from the above section provides the basis for SIoV parameters relationships, which are further used to develop the adaptation strategies for individual physical component (*i.e.*, subsystem) in this section. In SIoV every physical component is aware of its cyber entity by one-to-one mapping. As a result, any adaptation in the physical layer is notified to the cyber layer and *vice versa*. While designing the adaptation strategies, preferences are given to the locally known parameters over the global ones for every subsystem. Main objective of the adaptation techniques is to collect as much sensory data as possible while keeping the overall workload to an acceptable limit. Since SIoV is a cloud based cyber-physical system where all the subsystems are somehow communicating, hence dynamic adaptation of any subsystem may affect the entire system. There can be a range of adaptation strategies to choose from for every SIoV subsystem. Following sections describe some example adaptation strategies for the different SIoV subsystems. A comprehensive analysis of the adaptation strategies will be presented in a future article.

### 7.1. Vehicle Adaptation Strategy

Every vehicle has direct local knowledge of vehicle communication range ($r_{v}$), speed (*v*), and data rates ([λ]) over the vehicle arrival rate (ρ) and infrastructure inter-distance (*L*). As long as the vehicle workload ($\psi_{v}$) is below the threshold ($\psi_{v}^{th}$) level, data collection can increase ($\delta_{V}^{+}$), otherwise it is curtailed ($\delta_{V}^{-}$) (Algorithm 1).

In order to increase data collection ($\Theta^{+}$), additional safety information can be stored ($\lambda_{b}$). Other option is to increase the virtual communication range ($r_{v}$) to allow bigger platoons, so that more interactions can occur among the vehicles. On the other hand, data collection can be curtailed ($\Theta^{-}$) by reducing additional safety data ($\lambda_{b}$) and the communication range ($r_{v}$). If the infrastructure distance (*L*) is very long then the vehicles can elect ($\Theta^{*}$) leader of a platoon who will only handover the bulky message to the infrastructure. Such a setup will allow the vehicles to ignore some overhead interaction messages.

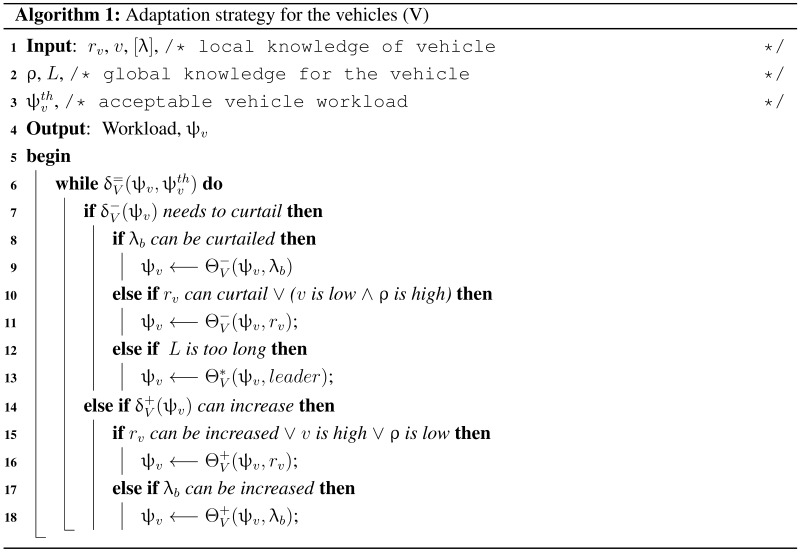

### 7.2. Infrastructure Adaptation Strategy

For the infrastructures $r_{i}$, ρ and *L* are the local knowledge and the objective of adaptation strategy is to balance ($\delta^{=}$) the infrastructure workload ($\psi_{I}$). As more sensory information ensure better understanding of the events hence more data collection ($\delta^{+}$) is preferable. Increasing $r_{v}$ range and collecting more safety information $\lambda_{b}$ is an automatic choice for more ($\Theta^{+}$) data collection. If workload needs to be curtailed ($\delta^{-}$) then traffic can be diverted to alternative routes, which reduces arrival rate (ρ). Also, $r_{v}$ reduction and leader election can reduce infrastructure workload (Algorithm 2).

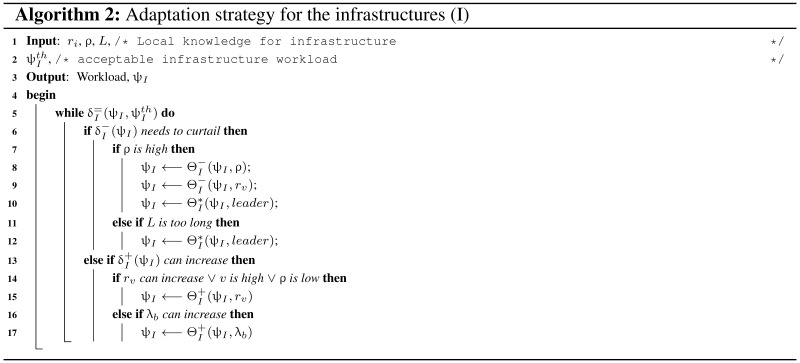

### 7.3. Home Adaptation Strategy

The number of friends ($n_{h}$) one home interacts at any time is related with the possible relationships. If $n_{h}$ is near the limit then the relationships can be prioritized and higher priority interactions will be given longer slots (Algorithm 3). The home subsystem can handover the sensory information more often and reduce the workload if the threshold level is very close. Another option can be, it works as a gateway for personal sensors of the vehicle owner. Personal sensors (e.g., Twitter/Facebook data) can improve the quality of the vehicle sensory data with additional human tagged details.

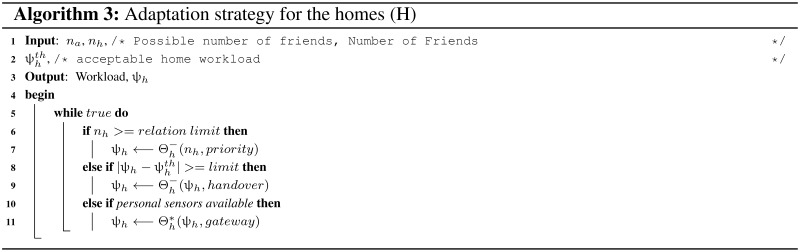

## 8. Conclusions and Future Works

Social Internet of Vehicles is a cloud based cyber-physical approach to tackle the internet of vehicles related issues in a more scalable and distributed manner. The cyber layer of the SIoV eases the integration of different IoT domains. Various relationship types and related policies open the opportunities of intelligent service compositions following social network rules. In this paper, we have designed the analytical models of the subsystems involved in the SIoV interaction process. The workload models for vehicle, infrastructure, home, and the cloud would help to understand the storage or computing requirements, which is vital for Big Data management. Cloud computing cost can be balanced for SIoV related applications deployment using workload model based dynamic adaptation. In this paper, we provide detailed simulation results of subsystem workloads at different parameter settings. Based on the characteristics analysis of SIoV, we find the system parameter relationships, which are further used to design example adaptation strategies for different SIoV subsystems. The proposed models would be useful to deploy SIoV based safety, efficiency or comfort applications.

Design and development of a robust cyber-physical IoV simulator that can incorporate adaptation strategies is our next future work. Also, integrating real vehicles with the simulated vehicles under the IoV simulator platform can offer interesting insights about deployment experience. Integration of heterogeneous IoT domains through the cyber layer is another possible work. Since vehicular sensory data is a key element of sensing-as-a-service model hence we are working on the development a multimedia sensory dataset for the IoV applications. The dataset can include both OBD based vehicular data and Smartphone sensor readings.
